# Young Carers in the Covid-19 Era: Characteristics, Pandemic Experiences, and Mental Health

**DOI:** 10.1192/bjo.2026.11064

**Published:** 2026-06-30

**Authors:** Lydia Ives, Tamsin Newlove-Delgado

**Affiliations:** University of Exeter, Exeter, United Kingdom

## Abstract

**Aims:**

Young carers (YCs), estimated to comprise 2–8% of the child and young person (CYP) population in England, face numerous sociodemographic challenges that put them at increased risk of poor mental health (MH). The Covid-19 pandemic exacerbated challenges by increasing caregiving responsibilities. However, research on YCs in the pandemic era remains limited. This study is a secondary analysis of the NHS England 2020 Mental Health of Children and Young People (MHCYP) survey, investigating the characteristics, pandemic experiences, and MH outcomes of YCs aged 11–22 in England.

**Methods:**

The NHS England MHCYP 2020 survey was a follow-up to the 2017 survey, taking place July/August 2020. The original 2017 cohort, selected through random probability sampling from the NHS Patient Register, included 9,117 CYP aged 2–19 and/or their parents. Of these, 3,570 (46%) completed the follow-up 2020 survey, now aged 5–22. The main measure of mental health in 2020 was the Strengths and Difficulties Questionnaire (SDQ), a broad measure of mental health difficulties. The 2020 survey included a range of questions about pandemic experiences, which were compared for YCs and non-YCs. Longitudinal regression analyses assessed whether YC status in 2017 predicted change in SDQ score between 2017 and 2020. Weighted analyses adjusted sequentially for baseline SDQ, demographics, and a comprehensive theoretically informed covariate set.

**Results:**

The analysis revealed some differences in demographic and pandemic-related variables between YCs and non-YCs. Parents of young carers were more likely to report that they could not afford food or had to use a food bank compared to parents of other young people (5.7% compared to 1.8%). Young carers were more likely than non young carers toreport that their household could not pay bills (5.5% compared to 1.8%). There was no evidence of difference in MH outcomes in 2020 as measured by the SDQ between YCs and non-YCs, nor did the longitudinal analysis indicate a greater deterioration in SDQ scores among YCs from 2017 to 2020.

**Conclusion:**

These findings suggest that YCs as a group were more likely to face socioeconomic challenges during the pandemic, although we found no differences in mental health outcomes. This may reflect protective factors at play or reflect study limitations. There was considerable attrition between the 2017 and 2020 surveys, requiring weighted analysis and reducing power. Further research is warranted to explore the complex interactions between caregiving, socioeconomic factors, and MH, and to develop risk-mitigation and mental health promotion strategies.

